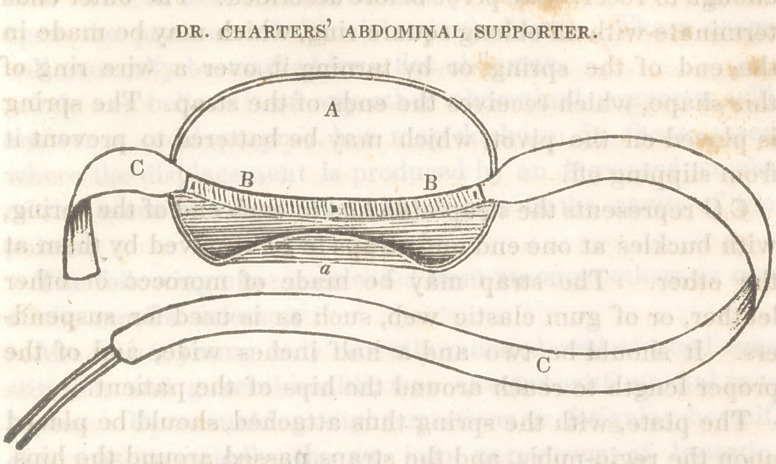# Observations on Prolapsus Uteri and Its Treatment; with an Account of Dr. Chartres’ Abdominal Supporter

**Published:** 1847-06

**Authors:** John Evans

**Affiliations:** Professor of Obstetrics and the Diseases of Women and Children, in Rush Medical College, Chicago, Ill.


ARTICLE III.

*Observations on Prolapsus Uteri and its Treatment; with an account of Dr. Charters' Abdominal Supporter.* By John Evans, M. D., Professor of Obstetrics and the Diseases of Women and Children, in Rush Medical College, Chicago, Ill.

Prolapsus Uteri is divided into three classes by the degree of the displacement: 1st. Partial; 2d. Incomplete; and 3d. Complete prolapsus.

The most common displacement of the uterus is what has been termed by authors partial prolapsus. It consists in a depression of the viscus below its natural position; the os tincæ descended but a little way into the vagina, and pressing its superior extremity forward into a larger fold than natural, just before its attachment to the cervix. This is liable to occur at all ages after puberty, while complete or even extensive prolapsion is seldom found in the female who has never been pregnant.

Incomplete prolapsus is a descent of the uterus so that the os tincæ approaches near to, or protrudes a little beyond the os externum. It is second in frequency.

Complete prolapsus is fortunately extremely rare, and consists in an inversion of the vagina, and the protrusion of the uterus in it without the labia. The forms of the disease differ pathologically only in degree.

The pathology of prolapsus uteri has been but imperfectly understood until quite recently; and in fact there is yet a veil of obscurity hanging over the subject.

Without hoping entirely to remove it, we will inquire into the condition of the parts in health, and the changes that are wrought by displacements of the uterus.

The uterus is situated near the centre of the pelvic cavity, and floats freely between the rectum posteriorly and the bladder before, with its fundus presenting upward, upon which rests the floating convolutions of the small intestines. Above and to the right is the cœcum of the colon, and to the left the segmoid flexure of this larger intestine, both of which float to some extent, and are liable to press down and rest upon it. On either side of the uterus are its only ligaments; the broad—simply a fold of the peritoneum—containing the others, all of which are loose, and afford no other support to the organ than to prevent great lateral inclinations or extensive rotations.

Below, it is received by the vagina, which is attached to and surrounds the cervix. Posteriorly, the vagina, for a distance, has no attachment, being lined exteriorly by the peritoneum, and here, of course, it assumes a membranous character. Anteriorly it is bound to the bas fond of the bladder by the vesico-vaginal septum, which can give it but little support, as the bladder is entirely loose and flexible at this point. The anterior and posterior walls of the vagina, being pressed in by the rectum and bladder are in contact, leaving the lateral walls narrow, which are bound by loose cellular tissue to the sides of the pelvis. Thus it is manifest that the superior portion of the vagina can afford but little support to the uterus. The vulvar extremity of the vagina is much more strongly bound by the lateral cellular attachments, the recto-vaginal and the urethro-vaginal septa. The vagina is situated in the curvilinear line of the common axis of the pelvis, and forms, at its attachment to the uterus, an angle of about sixty-five degrees with it. It rests upon the rectum and the perinæum, which are supported by the transversus peræneí and levator ani muscles, which form the floor of the pelvis and support its viscera.

In connection with this description of the parts, the position of the pelvis must be borne in mind. The plane of the superior strait being inclined forward and downward at an angle of about forty degrees, with the promontory of the sacrum projecting forward over its cavity, relieves the pelvic viscera and the perinæum, of much of the weight of the abdominal viscera and the force of the impulse given them downward by the contractions of the diaphragm, by throwing them forward upon the pubes and abdominal parieties. But at the same time it places the vagina more nearly in a vertical position, and thus places the uterus more directly over it.

The perinæum and the abdominal muscles just above the pelvis, are antagonists to the diaghragm and the superior abdominal parieties. This is a fact I do not recollect to have seen stated in this connection, of which any one may satisfy himself by placing his hands upon the abdomen above, and the regio-pubis, while making an effort at fæcecation. The regio-pubis will be found to project during the effort, and preventing it from doing it will materially interfere with the force upon the perinæum.

The relaxation, at least a fixed position without contraction, of the parieties of the lower part of the abdomen, to a certain extent, is necessary, to allow the force from above to press the abdominal viscera past the promontory of the sacrum into the pelvis.

Now when the system is debilitated, and the connections of the vagina are relaxed, the support to the pelvic viscera given by the transversus perinæi, levator ani, and the lower abdominal muscles being enfeebled, straining efforts at stool, in lifting, or other violent exercise, must tend directly to produce prolapsus. When from such causes the uterus is pressed downwards and forwards into the vagina, it is exceedingly difficult to induce it to resume its natural place—especially if the pressure of the abdominal viscera, the relaxation, or the efforts that caused it are yet operating.

For the symptoms of prolapsus the reader is referred to systematic authors.

The great frequency of the occurrence of a prolapsed condition of the uterus amongst the females of the west, must have attracted the attention of almost every physician in this region of country. And he has been extremely fortunate in practice who has not been much annoyed and perplexed in its treatment.

It is believed that displacements of the uterus are much more common in the western country than in any other section of the United States, for which there seems to be several good and satisfactory reasons found in the habits of life and want of general health amongst the sufferers.

The following causes, with others, operate to produce prolapsus uteri in almost all countries, but they are mentioned as being more common in the west.

1st. The female portion of our community are subjected to many more hardships than in older settled countries, and perform much more bodily exertion in walking, lifting, &c.

It is not unfrequently the case that women in the country labor in the field, reap grain, mow and pitch hay, plough, hoe, and in fact perform almost all the laborious duties so fitly assigned by the rules and customs of civilized sociey to the other sex. I know a widow lady who, with a family of daughters of robust constitution, carried on the farm with little if any help, except from them, for two or three years after the death of her husband. The consequence is, the girls have been for some time under treatment for prolapsus uteri. In the settlement of new countries there are always privations which impose upon females a necessity for greater bodily exertions than where the conveniences of life are abundant. This renders such exertions common, fixes public sentiment in favor of them, and they are carried to a much greater extent than necessity actually requires.

2d. They are subject to these oftentimes while the general health is impaired from the epidemic diseases of the country —intermittent and remittent fevers.

From what has already been said in reference to the pathology of prolapsus, the influence of general debility in predisposing to it, and rendering slight exciting causes most effective in its production, will be apparent.

In the summer and autumnal months the western country is usually visited by an epidemic of intermittent and remittent fevers, with a train of concomitant local affections either produced or modified by it. These often debilitate to a great extent, when appearing in their milder forms, without causing patients to entirely suspend their daily avocations. This is more particularly the case with females during these seasons of the year, when servants are scarce and often other members of the family, from sickness, require attention, and hence arises a fruitful cause of displacement of the uterus.

3d. The epidemic diseases often derange the functions of the uterus, producing engorgements while the parts are much relaxed, from the debility they produce.

The diseases of the west particularly affect the abdominal viscera. The congestions that attend every paroxysm of intermittent, spend their principal force upon the blood-vessels of the abdomen; hence we have enlargements of the spleen and liver, congestions of the portal circle, deranging the functions of the stomach and bowels; often causing the latter to become loaded with fœces, and engorgments of the uterus and its appendages. The load thus accumulated upon the pelvic viscera, together with their increased weight, can but have the effect to press them down and produce prolapsus.

4th. They frequently rise too soon after confinement and while yet feeble, enter upon the performance of the ordinary duties of housewifery.

I have thought that, from imprudence, and, in some cases, apparent necessity, the women of the west rise much earlier after confinement than in most other countries. This is a well known and operative cause of uterine displacements in all countries. The increased weight of the uterus, the relaxed condition of the vagina and perinæum, make it a matter of wonder that any female should rise in ten days or two weeks after delivery and escape the most troublesome prolapsus. Yet our western women often rise within the first week, and sometimes even within three or four days.

*Treatment.*—The treatment of prolapsus, as pursued, presents as many modes of practice as any other disease of the country. This arises from the diversity of opinion in reference to its pathology, the various degrees of prolapsus, and the variety of circumstances under which it occurs. And to a great extent, failures to relieve it depend upon a want of discrimination in making out the case and adapting the means.

The treatment is properly divided into the constitutional, local, and mechanical.

Of the constitutional treatment I do not propose to speak further than to say that it is important to relieve any disease under which the patient may be laboring by its appropriate remedies, as far as possible, and to restore the tone of the system. This is frequently exceedingly difficult, in consequence of the irritation arising from the displacement of the uterus produced by its nervous connections and extensive sympathies; and the debilitating leucorrhœa, which often attends it, sometimes as a cause and sometimes as a sequence. But generally an invigorating regimen and the use of tonics will be appropriate in the absence of inflammatory affections.

The local treatment must be pursued in connection with the general, and principally consists in astringent and tonic injections thrown into the vagina, bathing the vulva, &c.

The indications of mechanical treatment are to replace the uterus and retain it in its natural situation. These vary in the different degrees of displacement.

In the complete prolapsus the reduction is often difficult, and sometimes, in cases of long standing, impossible in consequence of adhesions. In incomplete it is effected with less difficulty. And in the partial generally very readily.

The operation is simply pressing the organ upward in the direction of the axis of the pelvis to its place.

The difficulty of filling the second indication is much greater, and has called forth the inventive genius of many physicians in devising apparatus.

Those in most general use are pessaries and abdominal supporters.

In complete prolapsus, after its reduction, the pessary properly applied, for a time at least, will be found the most successful. But it seldom effects a cure. The mode of its operation is such as to keep the vagina distended laterally and contracted longitudinally, and of course this will not serve to place the uterus in its proper situation, and strengthen the resistance to its descent into the vagina.

Pessaries are of various shapes and materials, of which I do not propose at length to speak, but will give my opinion in reference to the best, and the reasons for it. All those pessaries that depend upon their lodgment in the vagina, simply, for the support they give the uterus, although they may afford temporary relief from the descent of the uterus will do as much if not more harm than good eventually. The reason is plain—They distend the vagina, or they will not remain in situ, and of course cause it and its attachments to become relaxed to a greater extent than before, and thus weaken the strongest and almost the only natural support to the uterus. But there is a class of pessaries, to some extent although not entirely free from this objection—I mean stem pessaries. They consist of a bulb resembling the ordinary pessary, to which is attached a stem that protrudes from the vulva and is supported by a perineal compress or T bandage. In this the pessary may be small so as not greatly to distend the vagina, while the stem supports it and the uterus resting upon it, which can be so regulated in its length and attachment to the bandage as to retain the the uterus in the position desired.

The greatest objections to this are its distension, to some extent of the vagina, its unpleasantness to the patient, and the difficulty of introducing it; the latter of which has been almost entirely removed by an ingenious contrivance of Dr. Saunders, of Monrovia, Morgan county, Indiana, which is as follows: He takes a small gum elastic pessary, has a metallic stem made with the lower end suitably arranged for attachment to the T bandage, the shank smooth and round, of the proper length, with the superior end bifurcated so as the branches pass off in opposite directions, and almost at right angles with the shank. These branches he inserts into the pessary within the perforation on opposite sides. The shank will then fold down upon either edge of the pessary, and it can be introduced edgewise in the ordinary manner. Dr. Saunders, whose judgment and intelligence render him every way qualified to judge, has had an extensive practice with this instrument, and speaks highly of its usefulness. Another equally good, if not a better instrument, consists of a tube of gum elastic, of about the size and length of the vagina, made thin, filled with air, and hermetically sealed. This may be readily introduced, or removed for the purpose of being cleansed (a matter of much importance in all pessaries). It is light, and the only thing disagreeable or inconvenient about it is, that it requires to be kept in place by a compress on the vulva, retained by a T bandage. By the use of either of these, the complete or extensive, may be converted into a case of partial prolapsus. To relieve which it is necessary that the vagina, its attachments, and the perinæum should be allowed to support the uterus, for, without exercise they become weaker instead of stronger.

In partial prolapsus, the pessary does more harm than good, as I have had repeated opportunities of witnessing.

The abdominal supporter, in a certain class of cases is all that could be desired.

When the prolapsus is partial and the lower part of the abdomen prominent, it will generally afford immediate relief.

There are a host of different patterns for abdominal supporters—most of which have been patented by their inventors, and of the physicians, I may say disgracefully, for it tends to reduce them from the high stand they occupy in a liberal and scientific profession to the level of a mere handicraft or trade, to be pursued, not for the blessings it dispenses, but solely for the emoluments to be derived from it.

Many, no doubt unthoughtedly, have pursued this course; but they should remember the odium that attaches to the name of *Chamberlain*, who might have been ranked amongst the greatest benefactors, for the invention of the forceps had he not basely kept it secret, for the purpose of gain, until his name acquired a blot that neither the famed Letheon, nor even the waters of Lethe themselves, can ever wash away. There are, however, a few honorable exceptions, one of the best of which was invented by Wm. M. Charters, M. D., of Lebanon, Ohio. And as it answers all the purposes that can be derived from this class of instruments and possesses some advantages over all others, I most heartily recommend it to the profession. It is cheap and can be readily got up in any of our towns and villages in the country.

I had hoped, ere this, to have seen a full account of it from the pen of its accomplished inventor, given to the public. Shall we not yet hear from him?

The following cut will enable me to describe it so that physicians may understand it and have it manfactured.

DR. CHARTERS' ABDOMINAL SUPPORTER.

A *a* represents a plate made of tin, from six to seven inches long, to suit the patient. The object being to allow it as great a length as can be worn without reaching so near the superior anterior spinous process of the illeum on each side, as to cause irritation from pressure upon it. Its width is four inches. The edges being rounded as represented in the cut. The margin of the plate is wired to give it strength. The lower edge at *a* is turned up so as to project forward from a half to three fourths of an inch. At about two-thirds of the distance from the upper margin to the lower in the longitudinal centre of the plate, just above the margin that is rolled forward is a pivot, generally made of an ordinary stove-pipe rivet, and fastened there by passing the rivet through a small piece of tin and soldering it on with the head of the rivet resting against the plate. The end of the rivet should protrude one-third or half an inch. The plate is then swedged so as to give it a slight convexity at each end, on the back or side receiving the pivot. Thus prepared, it is covered with morocco, dressed buckskin, or velvet completely. The covering may be glued or pasted to the plate to make it fit smoothly.

B B represents a steel spring, six inches long, by one and a half inches wide, made thin enough to give, under moderate pressure; with a curvature, so that when the back or convex surface is laid on the plate, its ends will be about an inch above it. In the centre of this is a round hole, just large enough to receive the pivot before described. The outer ends terminate with an oblong square ring, which may be made in the end of the spring, or by turning it over a wire ring of this shape, which receives the ends of the strap. The spring is placed on the pivot, which may be battered to prevent it from slipping off.

C C represents the strap attached to each end of the spring, with buckles at one end and straps to be received by them at the other. The strap may be made of morocco or other leather, or of gum elastic web, such as is used for suspenders. It should be two and a half inches wide, and of the proper length to reach around the hips of the patient.

The plate, with the spring thus attached, should be placed upon the regio-pubis, and the straps passed around the hips, and buckled so as to afford the required amount of pressure.

This will support the abdominal viscera, and by antagonising with the promontory of the sacrum, prevent to a great extent, the impulses otherwise given to their pressure down upon the uterus.

From what has already been said in reference to the mechanism of these parts, it will be seen to be particularly appropriate in cases where there is much tumefaction of the abdomen, from an increase in size of its contents.

This instrument has an advantage over all those that depend upon a spring passing around the hip, in that it can be worn in any position and more comfortably, it is more readily adapted to the patient, and can be regulated in the amount of pressure to suit any case.

It has an advantage over those that consist simply in pads and straps in that it makes greater pressure directly over the pubes, the point where it is required; and over all others in that it can be readily got up in any part of the country, in a short time, and at a moderate expense—say, from one dollar to two and a half, or more, according to the style.

I have been using this instrument for the last four or five years, and with the most satisfactory results, in almost all cases of partial prolapsus, whether it was the original condition of the case, or was that following an extensive displacement partially relieved, as herein before mentioned. In some cases where I have tried it, it did not seem to answer well, in consequence of the prolapsus not depending upon the pressure of the abdominal viscera. Those cases will generally be marked by a flat abdomen.

I do not believe it or any other abdominal supporter will answer a good purpose, but may do harm, in those cases where the displacement is produced by an increased weight of the uterus itself, or from any tumor in the cavity of the pelvis.

The following case is selected from amongst others as one of the most satisfactory:

Mrs. S., a young lady recently married and of good constitution and general health, was affected with partial prolapsus. Upon making slight exertions or fatiguing herself, she was repeatedly thrown into paroxysms of hysteria, which, by the ordinary remedies and a few days' rest in the recumbent position would subside. These attacks were frequent during eighteen months, when I applied Dr. Charters' supporter, which gave the most perfect relief. Having occasion, some time after, to use the instrument she was wearing as a pattern for the workmen to make others by, she assured me that she was rendered miserable by its absence, but when replaced was entirely relieved.

There is a point in the management of prolapsus in child bearing women too generally over-looked or neglected by physicians—it is the favorable opportunity afforded at confinement for effecting a cure.

The great change effected in the condition of the parts by pregnancy, renders it a most favorable time while they are resuming their natural and quiescent condition, to restore them to that position and tone which will be a cure of the displacement. Such patients should be rigidly confined to the recumbent position while the abdomen is properly supported until the parts have regained their natural size and relation.

## Figures and Tables

**Figure f1:**